# Atlantic Cod Piscidin and Its Diversification through Positive Selection

**DOI:** 10.1371/journal.pone.0009501

**Published:** 2010-03-02

**Authors:** Jorge M. O. Fernandes, Jareeporn Ruangsri, Viswanath Kiron

**Affiliations:** Faculty of Biosciences and Aquaculture, Bodø University College, Bodø, Nordland, Norway; Providence Health Care, Canada

## Abstract

Piscidins constitute a family of cationic antimicrobial peptides that are thought to play an important role in the innate immune response of teleosts. On the one hand they show a remarkable diversity, which indicates that they are shaped by positive selection, but on the other hand they are ancient and have specific targets, suggesting that they are constrained by purifying selection. Until now piscidins had only been found in fish species from the superorder Acanthopterygii but we have recently identified a piscidin gene in Atlantic cod (*Gadus morhua*), thus showing that these antimicrobial peptides are not restricted to evolutionarily modern teleosts. Nucleotide diversity was much higher in the regions of the piscidin gene that code for the mature peptide and its pro domain than in the signal peptide. Maximum likelihood analyses with different evolution models revealed that the piscidin gene is under positive selection. Charge or hydrophobicity-changing amino acid substitutions observed in positively selected sites within the mature peptide influence its amphipathic structure and can have a marked effect on its function. This diversification might be associated with adaptation to new habitats or rapidly evolving pathogens.

## Introduction

Innate immunity is considered to be particularly important in teleosts, since their adaptive immune response has poor immunological memory and a short-lived secondary response [1]. Moreover, it is limited by environmental constraints such as temperature [2]. Amongst the various components of the teleost innate immune system, antimicrobial peptides (AMPs) are thought to play a crucial role as a first-line of host defence against potential pathogens [3]. AMPs are a group of small peptides with broad-spectrum activity that show remarkable diversity in sequence, secondary structure and function. Nevertheless, they are generally cationic and amphipathic, which allows them to interact with cell membranes and form pores by barrel-stave, carpet or toroidal pore mechanisms [4]. In teleosts, several AMPs have been identified in lymphoid organs, epithelial tissues, liver and blood [5], [6], [7], [8], [9], [10].

Piscidins constitute a novel family of AMPs found in teleosts, which display broad-spectrum activity against bacteria [10], fungi [11] and even viruses [12]. The piscidin gene consists of three introns and four exons that code for a putative precursor comprising a hydrophobic signal peptide, the mature piscidin and a carboxy-terminal prodomain [13]. Most piscidins are linear peptides with less than 26 residues and a high proportion of basic amino acids, phenylalanine and isoleucine. Based on similarities in gene structure and primary sequence, it has been suggested that piscidins are closely related to pleurocidins, a family of AMPs found in flatfish [3], [13]. First identified in the mast cells of the hybrid striped bass [10], piscidins are known to be widespread amongst Perciformes. They have been detected by immunohistochemistry in fish from several families, including the Moronidae, Sciaenidae, Serranidae, Cichlidae, Siganidae and Belontidae [14]. A gene coding for a piscidin-like peptide has also been cloned in a Gasterosteiforme, the spotted seahorse (*Hippocampus kuda*) (AY864343).

Teleosts are the most numerous group of extant vertebrates, inhabiting different niches and at increased risk of exploitation by a wide range of potential pathogens. Unlike other marine vertebrates, which have an external layer of keratinised skin that constitutes an efficient natural barrier, fish are less protected against invading pathogens, since the epidermal surface of their skin is composed mostly of living cells with minimal keratinisation [15]. The piscidin genes of teleosts have to cope with this diversity of rapidly evolving pathogens and it is therefore likely that they are shaped by positive (diversifying or adaptive) selection. On the other hand, it is also plausible that piscidin can be under negative (purifying) selection, since its targets are quite specific (eg, the cell membrane). In protein coding genes, the ratio (ω) between nonsynonymous (amino acid-changing, dN) and synonymous (silent, dS) substitution rates is related to evolutionary constraints at the protein level [16]. A value of ω>1 indicates positive Darwinian selection, whereas ω<1 suggests negative selection. In the present study, we have identified piscidin in Atlantic cod (*Gadus morhua*), a more basal teleost of the Paracanthopterygii superorder, and examined nucleotide divergence in all piscidin genes reported to date and used likelihood methods with various models of evolution to investigate patterns of positive selection.

## Materials and Methods

### Ethics Statement

All animal handling protocols were in accordance with the national guidelines detailed in the “Norwegian Regulation on Animal Experimentation” Act and the experiments were approved by the National Animal Research Authority (Forsøksdyrutvalget, Norway).

### Biological Samples

Two-year old *G. morhua* were kept at the Mørkvedbukta research station of Bodø University College (Norway) in 2000 litre fibre glass tanks equipped with a flow-through sea water system (temperature range 7–8°C) and under a 12∶12 photoperiod regime. The fish were fed daily *ad libitum* with a commercial diet (BioMar, Norway). Two days after stimulation with heat killed *Vibrio anguillarum*, the fish were humanely killed by immersion in an anaesthetic bath containing of 3-aminobenzoic acid ethyl ester (0.6 g·l^−1^, Sigma). Head kidney samples were collected, snap frozen in liquid nitrogen and kept at −80°C for subsequent RNA extraction.

### *In silico* Analysis and Piscidin Cloning

DNA sequences of piscidin and piscidin-like peptides from different teleost species were obtained from the NCBI database (http://www.ncbi.nlm.nih.gov/), translated and used as probes to screen the Codgene database (http://ri.imb.nrc.ca/codgene/) with TBLASTN and TBLASTX algorithms. The retrieved expressed sequence tags were assembled with CAP3 (http://pbil.univ-lyon1.fr/cap3.php) and a single contig representing a possible candidate for Atlantic cod piscidin was generated. This sequence was used to design the following forward and reverse primers, respectively: 5′-GTATCTGAAAGGATGAGGTATATTG-3′ and 5′-CTTGAAACATGCGAACTGC-3′.

Total RNA was extracted from 100 mg of head kidney as previously reported [17]. Following treatment with the gDNA wipeout buffer (Qiagen) to remove potentially contaminating genomic DNA, RNA quality was assessed by agarose gel electrophoresis and its quantity determined using a Nanodrop spectrophotometer (Nanodrop Technologies/Saven Werner, Norway). cDNA was synthesized with the QuantiTect reverse transcription kit (Qiagen) following the manufacturer's protocol. Piscidin was amplified from cDNA by PCR using the primers above and the following thermocycling parameters: initial denaturation at 95°C for 6 min, 36 cycles of denaturation at 95°C for 30 sec, annealing at 56°C for 30 sec and extension for 1 min at 72°C, with a final elongation at 72°C for 10 min. The amplicon was excised from the gel with the QIAquick gel extraction kit (Qiagen), cloned onto a TA vector and sequenced as described elsewhere [17].

### Sequence Analysis

Experimental sequence data were analysed and assembled with CodonCode Aligner (http://www.codoncode.com/aligner) and the contig containing good quality sequences was compared with the sequences available at the non-redundant database at NCBI using BLASTX. The piscidin nucleotide sequences were translated using DNAMAN (Lynnon Biosoft, Canada) and the putative proteins aligned with the CLUSTALW algorithm using a BLOSSUM matrix with the default parameters (http://npsa-pbil.ibcp.fr). Pair-wise protein sequence comparisons were performed with BioEdit [18].

### Phylogenetic Inference

Piscidin phylogenies were reconstructed using maximum likelihood (ML) and Bayesian inference methods using PhyML [19] and MrBayes v3.1.2 [20], respectively. Complete coding sequences (CDS) of the 10 piscidin genes reported to date from 9 teleost species (Table 1) were first aligned with ClustalW using BioEdit [18]. This nucleotide alignment was then used to generate a codon alignment using the software available at the HCV server (http://hcv.lanl.gov/). The C-terminal portion of the codon aligned piscidin sequences was too variable and hence not included in the positive selection tests. Instead, a codon alignment comprising 86.5% of the total CDS and without stop codons (Figure S1) was used. The best models of nucleotide substitution were selected with MrModelTest v2.3 (available from www.abc.se/~nylander/) and PAUP v4.0b10 [21]. The SYM+G model selected by the hierarchical likelihood ratio test (LRT) was subsequently found to be more appropriate than the GTR+I model suggested by the Akaike information criteria and was therefore chosen as the model of sequence evolution in the Bayesian analysis, using the following parameters: Lset nst = 6 rates = gamma; Prset statefreqpr = fixed(equal). Four Markov chains were run for 5·10^5^ generations sampling every 100 generation and a consensus tree was built after burning 1,250 trees.

**Table 1:** Genbank accession numbers of piscidin sequences from teleosts.

Species	Gene	Accession
Paracanthopterygii		
Gadiformes		
Gadidae		
* Gadus morhua*	Piscidin	FJ917596
Acanthopterygii		
Gasterosteiformes		
Syngnathidae		
* Hippocampus kuda*	Plp	AY864343
Perciformes		
Sinipercidae		
* Siniperca chuatsi*	Moronecidin	AY647433
Moronidae		
* Dicentrarchus labrax*	Dicentracin	AY303949
* Morone chrysops*	Piscidin-1	AF394243
* Morone saxatilis*	Piscidin-2	AF394244
Serranidae		
* Epinephelus akaara*	Piscidin	EU741828
* Epinephelus coioides*	Epinecidin	EU741829
	Piscidin	AY294407
Sciaenidae		
* Larimichthys crocea*	Piscidin	EU741827

### Tests of Selection

Differences in sequence diversity between the regions that code for the signal peptide and mature piscidin were ascertained by calculating the average number of synonymous and nonsynonymous substitutions, insertions and deletions in the codon alignments using SNAP [22]. This algorithm is based on the method developed by Nei and Gojobori [23] and it performs pairwise comparisons of all sequences in an alignment.

The hypothesis of positive selection was tested using the ML methods implemented in the CODEML program of PAML v4.2 [24], [25] and in the HyPhy software from the Datamonkey cluster [26]. In PAML, the data set was fitted to 6 models of codon substitution [16]: M0 (one ratio), fitting a single value of ω across all sites; M1, which allows for two site classes (ω = 1, 0<ω<1); M2 (positive selection) with three site classes (ω = 1, 0<ω<1 and ω>1); M3 (discrete), which has three discrete site classes with different ω values; M7 (β), which assumes a β distribution of class sites that does not allow for selection (0<ω<1); and M8 (continuous), which is similar to M7 but has an additional class with ω>1. Bayesian posterior probabilities were calculated for positively selected sites using naïve empirical Bayes (NEB) for model M3 or Bayes empirical Bayes (BEB) in the case of models M2 and M8. LRTs were performed to compare the corresponding models with and without selection (ie, M2 versus M1, M3 versus M0 and M8 versus M7). Statistical significance is determined by comparing twice the log-likelihood scores (2ΔLnL) to a χ^2^ distribution with degrees of freedom equal to the difference in the number of parameters between the models to be compared [24].

For the HyPhy analysis, the random effects likelihood (REL) model of molecular evolution was used. REL is a generalised version of the site by site positive selection analysis implemented in PAML and it is the most powerful of the three methods (SLAC, FEL and REL) included in HyPhy [26]. As implemented in Datamonkey, REL fits a 3×3 matrix of dN and dS rates, thus allowing dS and dN to vary across sites independently. The piscidin data set was analysed using the nucleotide substitution model 001001 with an AIC of 1799.95.

### Structure Prediction

A Schiffer-Edmundson representation of piscidin from *G. morhua* was obtained with the Helical Wheel Projections software available at http://rzlab.ucr.edu/scripts/wheel/wheel.cgi.

## Results and Discussion

**Figure 1:**
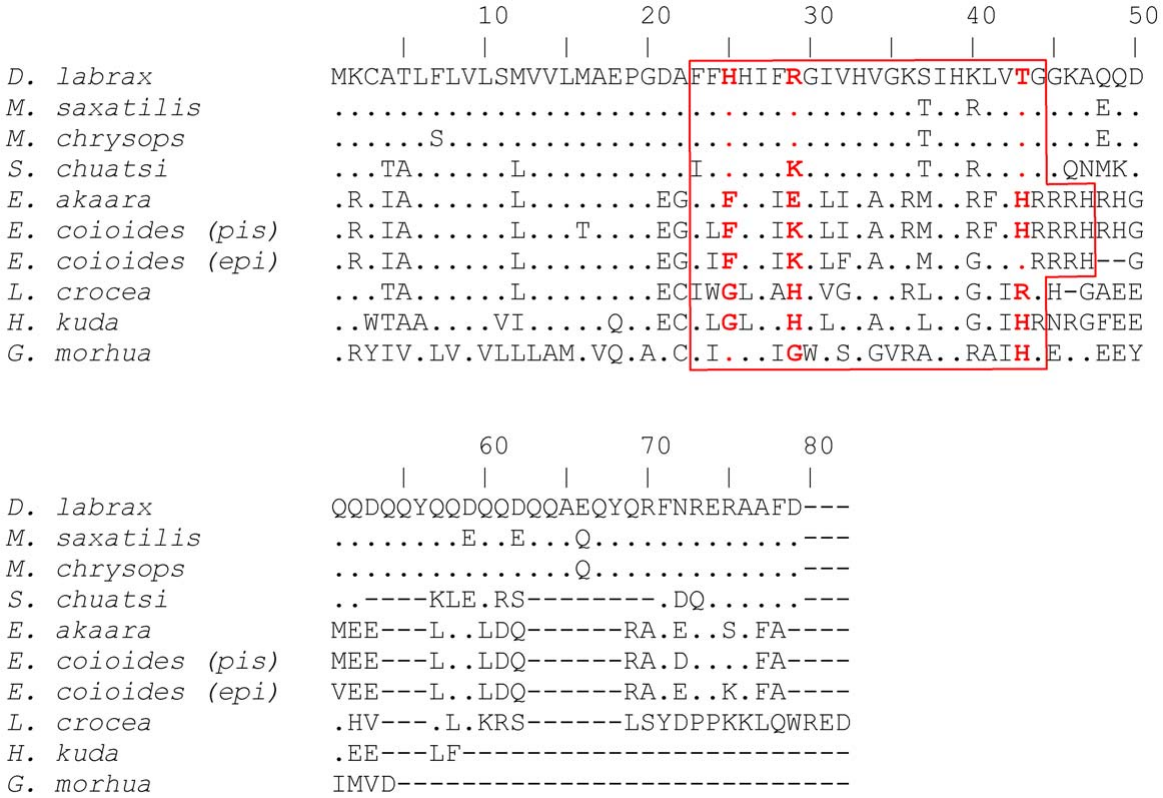
ClustalW multiple sequence alignment of putative piscidin peptides from teleosts. Amino acid residues identical to *D. labrax* piscidin are represented by a dot and alignment gaps are indicated by a dash. The mature peptides are boxed and positively selected residues are highlighted in bold red.

A comprehensive screen of the Codgene database retrieved three unidentified expressed sequence tags (accessions: all_v2.0.10048.C1, sb_gmnlkras_0002k16.t7 and sb_gmnlkfas_0004j05.t7) that shared 36% identity with the moronecidin precursor from the striped bass (*Morone saxatilis*) (AF385583). Using specific primers based on the contig of these sequences, we have isolated a 278 bp cDNA that contained the full length CDS of a piscidin orthologue in Atlantic cod (FJ917596).The putative mature cod piscidin is rich in isoleucine (27.3%) and histidine (22.7%) and it contains the two conserved histidines that define the piscidin group of antimicrobial peptides (Figure 1). This result shows that piscidins are an ancient family of host defence peptides not restricted to higher teleosts (ie, Acanthopterygii) as hitherto thought, since *G. morhua* is a basal teleost of the Paracanthopterygii superorder [27].

Most piscidin genes identified to date code for a precursor comprising a 22-residue signal peptide, a mature (active) peptide of 22–25 residues and a variable C-terminal region [3]. The putative 54-residue *G. morhua* piscidin precursor also shares this structure but shows a relatively low overall conservation at the amino acid level (Figure 1). The signal peptide has a relatively high identity between orthologues (Figure 1; Table S1), ranging from 54.5% to 100% between any species of the Acanthopterygii superorder. In contrast, the *G. morhua* sequence is very different from any other fish species and amino acid identities with its orthologues range from 27.2% to 36.3%. This marked divergence may be explained by the longer evolutionary distance between *G. morhua* (Paracanthopterygii) and the other fishes. The mature piscidin is generally less conserved across the different taxa, sharing only 31.8% to 95.4% identity between species at the amino acid level (Figure 1; Table S2). In particular, the *G. morhua*, large yellow croaker (*Larimichthys crocea*) and *H. kuda* piscidins are poorly conserved. A difference in conservation levels amongst various domains is common amongst AMPs. The cathelicidin family is a striking example of this feature. Cathelicidins are only recognised by their conserved signal sequence, since the mature peptides are diverse in length, amino acid sequence and even secondary structure [28].

**Figure 2:**
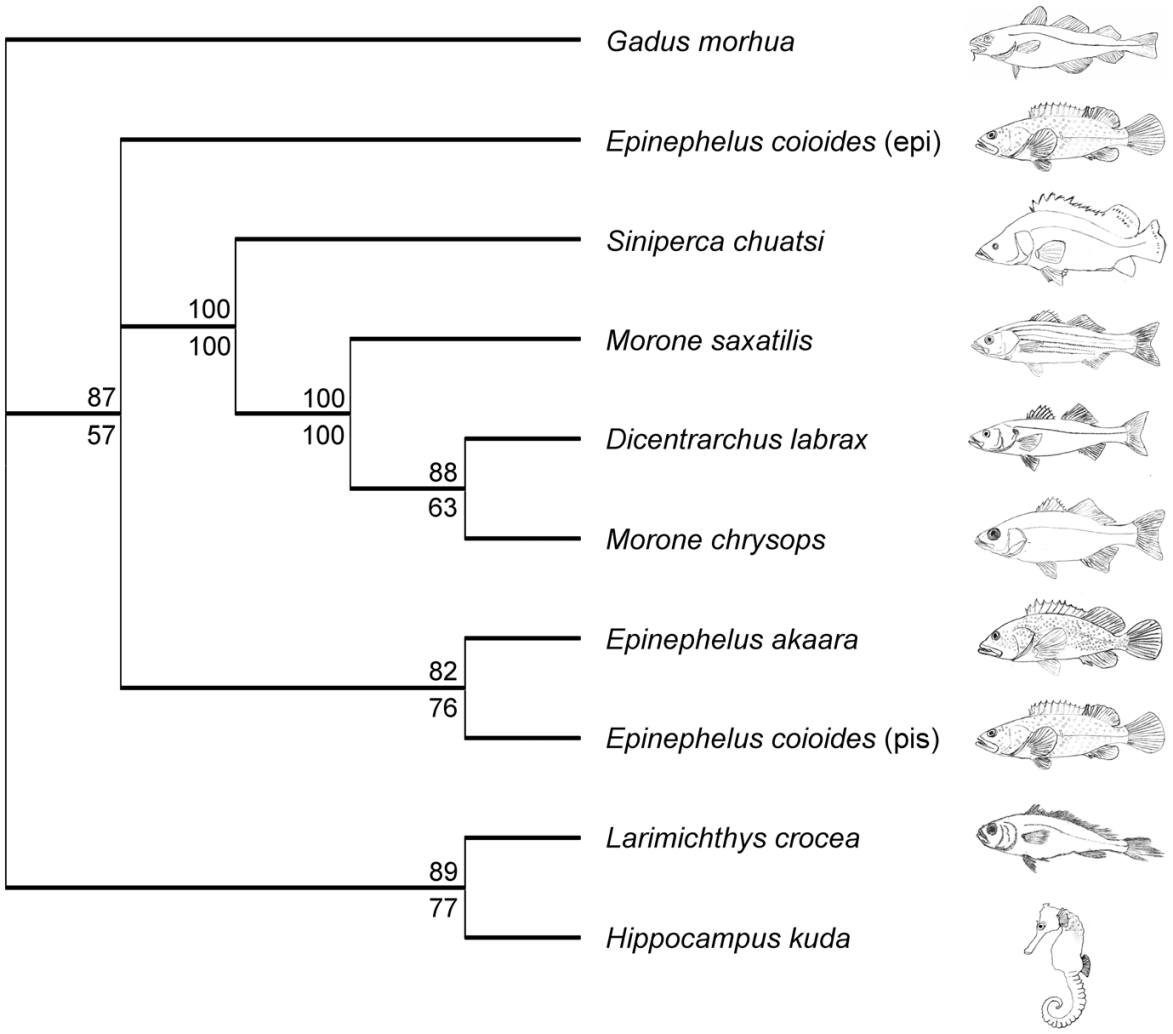
Unrooted rectangular cladogram illustrating the phylogenetic relationship between piscidins. The SYM+G model was selected for the Bayesian analysis and the consensus tree was built after burning 1,250 trees from the 5·10^5^ generations. The likelihood phylogeny was obtained with a HKY nucleotide substitution model with a discrete γ distribution (4 categories, γ shape parameter 2.0) and 100 bootstrap data sets. Bayesian posterior probabilities and maximum likelihood bootstrap values are indicated as percentages above and below the tree nodes, respectively.

Both the ML and Bayesian inference produced the same phylogenetic tree with high credibility support (Figure 2). In general, the topology of this tree is in accordance with the currently accepted taxonomic relationships amongst the various fishes. The exceptions are *L. crocea* and *H. kuda* piscidins, which cluster together and separately from the other Acanthopterygii. The complete phylogenetic reconstruction including branch lengths (Figure S2) was used for the PAML and HyPhy analyses of diversifying selection.

The site-specific analyses in PAML identified positively selected sites in the piscidin gene and LRTs revealed that models which allowed for adaptive selection fitted the data better than those which did not (M3 versus M0, p = 0; M8 versus M7, p = 0.04) (Table 2). This result is in agreement with previous reports showing that positive selection is contributing for the accelerated rate of amino acid substitutions in the coding regions of other fish antimicrobial peptides, including hepcidin [29] and pleurocidin [30]. Unfortunately, the positively selected sites in pleurocidin were not identified. In this study, the Bayesian approach in model M3 revealed three positively selected sites at positions 25 (p = 0.97, where *p* denotes posterior probability of assigning a site to the positively-selected class), 29 (p = 1.00) and 43 (p = 0.99), whereas only position 29 was found to be under adaptive selection according to model M8 (p = 0.95). Model M8 in PAML uses BEB, which is more suited for smaller data sets [31], and should therefore be considered the most reliable result.

**Table 2:** Identification of positively selected sites in piscidins by maximum likelihood analysis using various models of evolution.

Method	Model	Parameter estimates	Ln Likelihood	Model comparison	Positively selected sites a
CODEML	M0: neutral	ω = 0.28	−809.60		None
	M1: nearly neutral	ω_0_ = 0.14, ω_1_ = 1.00p_0_ = 0.67, p_1_ = 0.33	−794.42		Not allowed
	M2: positive selection	ω_0_ = 0.15, ω_1_ = 1.00, **ω_2_ = 13.76**p_0_ = 0.66, p_1_ = 0.31, p_2_ = 0.03	−793.20	M2 vs M12ΔLnL = 2.44, df = 2, p = 0.29	29
	M3: discrete	ω_0_ = 0.05, ω_1_ = 0.29, **ω_2_ = 2.25**p_0_ = 0.22, p_1_ = 0.59, p_2_ = 0.19	−792.42	M3 vs M02ΔLnL = 34.36, df = 4, p = 0.00	25, **29**, 43
	M7: β	p = 0.73, q = 1.27	−795.43		Not allowed
	M8: β + ωS>1	p = 0.73, q = 1.27**ω = 2.31**p_0_ = 0.83, p_1_ = 0.17	−792.29	M8 vs M72ΔLnL = 5.68, df = 2, p = 0.04	29
HYPHY	REL	**ω = 1.14**			25, **29**

a Only positively selected sites with Bayesian posterior probabilities equal or greater than 95% are indicated. Sites with a posterior probability greater than 99% are highlighted in bold.

One positively selected site at codon 29 was also identified by BEB in M2 but this model was not significantly better than the nearly neutral model M1 (p = 0.29, Table 2). The REL algorithm in HyPhy showed that piscidin codons 25 and 29 are under adaptive selection (p = 0.99 and p = 1.00, respectively). In spite of the high level of consistency between the PAML and HyPhy methods, there is one discrepancy related to position 43, which was only identified by model M3 in PAML. This might be due to variation in synonymous mutation rate, which is not accounted for by CODEML and can therefore lead to false positives. All positively selected sites were found only in the mature peptide and not in the signal peptide (Figure 1). Codon 29, which was found to be a positively selected site with statistical support and consensus by models M3, M8 and REL, is particularly variable (R, K, E, H or G).

**Figure 3:**
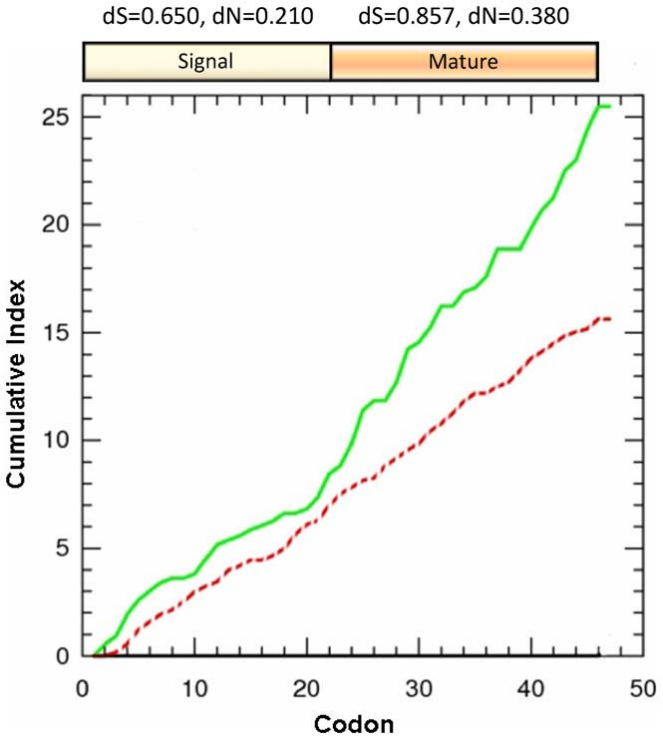
Average synonymous versus nonsynonymous nucleotide substitutions in piscidins. Cumulative nonsynonymous (solid green line) and synonymous (dashed red line) substitutions for all pairwise comparisons are plotted along the first 46 residues of the piscidin precursor. Generally the number of nonsynonymous substitutions exceeds the synonymous replacements throughout the coding sequence. Divergence at nonsynonymous sites is higher in the mature peptide region. dN and dS values for the signal and mature peptides are also indicated above the corresponding regions.

The average ω for all pairwise comparisons between piscidin sequences was 0.289 (dN = 0.285, dS = 0.987), indicating overall purifying selection that is probably due to functional constraints [32]. This shows that a disproportionately higher number of changes occur at synonymous sites than expected by chance. A closer inspection revealed significant differences in dN throughout the CDS: it is 81% higher in the region that codes for the mature peptide than in the signal sequence (Figure 3; p = 0.023, Wilcoxon signed rank test). Similarly, dS shows a 32% increase in the mature piscidin compared to the signal peptide (Figure 3; p<0.001, Wilcoxon signed rank test). These differences in substitution rates indicate that the selective pressure shaping piscidin is not uniform throughout its CDS. The hyperdivergence in the mature peptide region might be explained by an elevated mutation rate, as reported in other vertebrate antimicrobial peptides [33], [34]. It is noteworthy that divergence is highest at the end of the first exon, which codes not only for the N-terminal part of the mature piscidin but also for the less divergent signal sequence.

**Figure 4:**
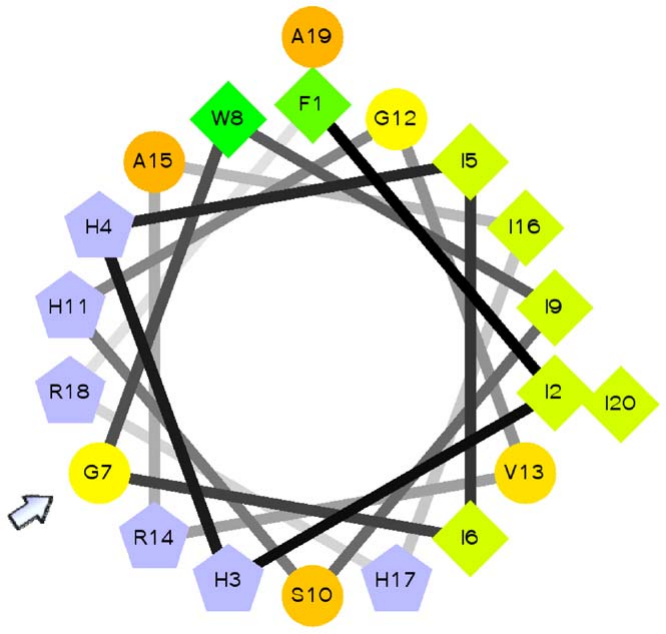
Amphipathic α-helical structure of cod piscidin. Schiffer-Edmundson helical wheel projection of piscidin from *Gadus morhua*, showing that it is predicted to adopt an almost perfect amphipathic structure. The amphipathicity of the helix is disrupted by glycine at position 7 (arrow), which corresponds to a positively selected site. Hydrophilic charged and very hydrophobic residues are represented by grey pentagons and green diamonds, respectively. The circles denote other neutral or polar amino acids. Residues are numbered starting from the amino terminus of the mature peptides.

Since all positively selected sites in the piscidin gene are located within the region corresponding to the mature peptide, we investigated how nonsynonymous substitutions at these codons might influence its secondary structure. In dodecylphosphocholine or sodium dodecylsulfate micelles, piscidin-1 from *M. chrysops* adopts an α-helical conformation [35], [36]. This α-helix has an almost perfect amphipathic structure, where hydrophobic and hydrophilic residues are placed in either side of the helix. The high positive net charge at physiological pH and the amphipathic nature of piscidin-1 are thought to be crucial for its ability to permeabilise bacterial membranes, probably through the formation of toroidal pores [35]. Several amino acid substitutions observed in positively selected sites within the mature peptide change its charge or amphipathicity. In particular, site 29 was identified by all models as being positively selected. In *G. morhua* piscidin this position corresponds to glycine, whereas in all other piscidins it is replaced by a basic residue. The presence of glycine at this position disrupts the amphipathic nature of the peptide (Figure 4), which in turn might have a profound effect on its function. We are currently characterizing *G. morhua* piscidin to investigate the influence of these nonsynonymous substitutions in the structural and functional divergence of piscidins. The positively selected sites identified in the gene section that codes for the mature peptide are likely to be associated with the adaptation of piscidin to pathogens found in new ecological niches and/or the need to protect the host against rapidly evolving pathogens.

## Supporting Information

Table S1 Percentage identity matrix of the signal peptide of piscidin in different teleost species.
(0.04 MB DOC)

Table S2 Percentage identity matrix of the mature piscidin sequences from different teleost species.
(0.04 MB DOC)

Figure S1 (A) Codon aligned multiple sequence alignment of piscidin nucleotide sequences corresponding to signal and mature peptides (46 codons). The pro-domain was too divergent to be included in the maximum likelihood analyses of positive selection. (B) ClustalW multiple sequence alignment of the corresponding putative peptides.
(1.44 MB TIF)

Figure S2 Reconstruction of piscidin phylogeny. This phylogram was constructed using Bayesian and likelihood methods. The SYM+G model was selected for the Bayesian analysis and the consensus tree was built after burning 1,250 trees from the 5•105 generations. The likelihood phylogeny was obtained with a HKY nucleotide substitution model with a discrete γ distribution (4 categories, γ shape parameter 2.0) and 100 bootstrap data sets. Bayesian posterior probabilities and maximum likelihood bootstrap values are indicated as percentages above and below the tree nodes, respectively. The scale bar indicates distance (branch length).
(1.04 MB TIF)
