# Supplementary material for: Atlantic Cod Piscidin and Its Diversification through Positive Selection
Source: PLoS One. 2010 Mar 2;5(3):e9501. doi: 10.1371/journal.pone.0009501 (PMC2830478; doi:10.1371/journal.pone.0009501)
Supplement: Table S2 — Percentage identity matrix of the mature piscidin sequences from different teleost species. (0.04 MB DOC) [file pone.0009501.s002.doc]

**Table S2.** Percentage identity matrix of the mature piscidin sequences from different teleost species.

|  | Gm_FJ917596 | Sc_AY647433 | Dl_AY303949 | Ms_AF394244 | Mc_AF394243 | Ea_EU741828 | Ec_EU741829 | Ec_AY294407 | Lc_EU741827 | Hk_AY864343 |
| --- | --- | --- | --- | --- | --- | --- | --- | --- | --- | --- |
| Gm_FJ917596 | ID |  |  |  |  |  |  |  |  |  |
| Sc_AY647433 | 40.9 | ID |  |  |  |  |  |  |  |  |
| Dl_AY303949 | 40.9 | 81.8 | ID |  |  |  |  |  |  |  |
| Ms_AF394244 | 45.4 | 90.9 | 90.9 | ID |  |  |  |  |  |  |
| Mc_AF394243 | 40.9 | 86.3 | 95.4 | 95.4 | ID |  |  |  |  |  |
| Ea_EU741828 | 40.0 | 40.0 | 40.0 | 44.0 | 40.0 | ID |  |  |  |  |
| Ec_EU741829 | 40.0 | 40.0 | 36.0 | 40.0 | 36.0 | 92.0 | ID |  |  |  |
| Ec_AY294407 | 32.0 | 48.0 | 48.0 | 48.0 | 48.0 | 72.0 | 76.0 | ID |  |  |
| Lc_EU741827 | 31.8 | 45.4 | 40.9 | 40.9 | 40.9 | 28.0 | 28.0 | 32.0 | ID |  |
| Hk_AY864343 | 31.8 | 45.4 | 50.0 | 50.0 | 50.0 | 44.0 | 48.0 | 52.0 | 59.0 | ID |

Dl, *Dicentrarchus labrax*; Ea, *Epinephelus akaara*; Ec, *Epinephelus coioides*; Gm, *Gadus morhua*; Hk, *Hippocampus kuda*; Lc, *Larimichthys crocea*; Mc, *Morone chrysops*; Ms, *Morone saxatilis*; Sc, *Siniperca chuatsi*.
