# Supplementary figures and images for: Atlantic Cod Piscidin and Its Diversification through Positive Selection
Source: PLoS One. 2010 Mar 2;5(3):e9501. doi: 10.1371/journal.pone.0009501 (PMC2830478; doi:10.1371/journal.pone.0009501)

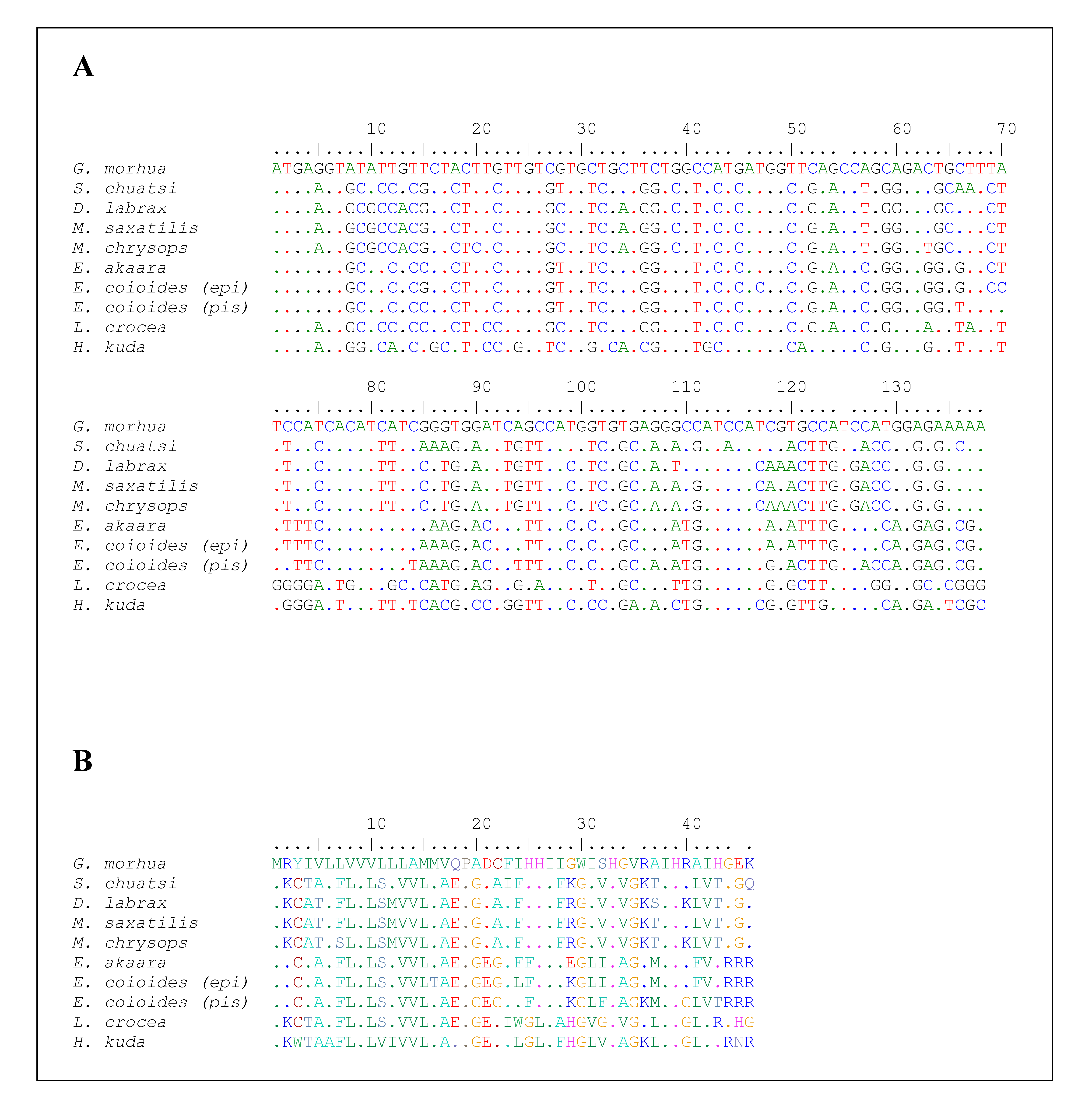

Supplement: Figure S1 — (A) Codon aligned multiple sequence alignment of piscidin nucleotide sequences corresponding to signal and mature peptides (46 codons). The pro-domain was too divergent to be included in the maximum likelihood analyses of positive selection. (B) ClustalW multiple sequence alignment of the corresponding putative peptides. (1.44 MB TIF) [file pone.0009501.s003.tif]

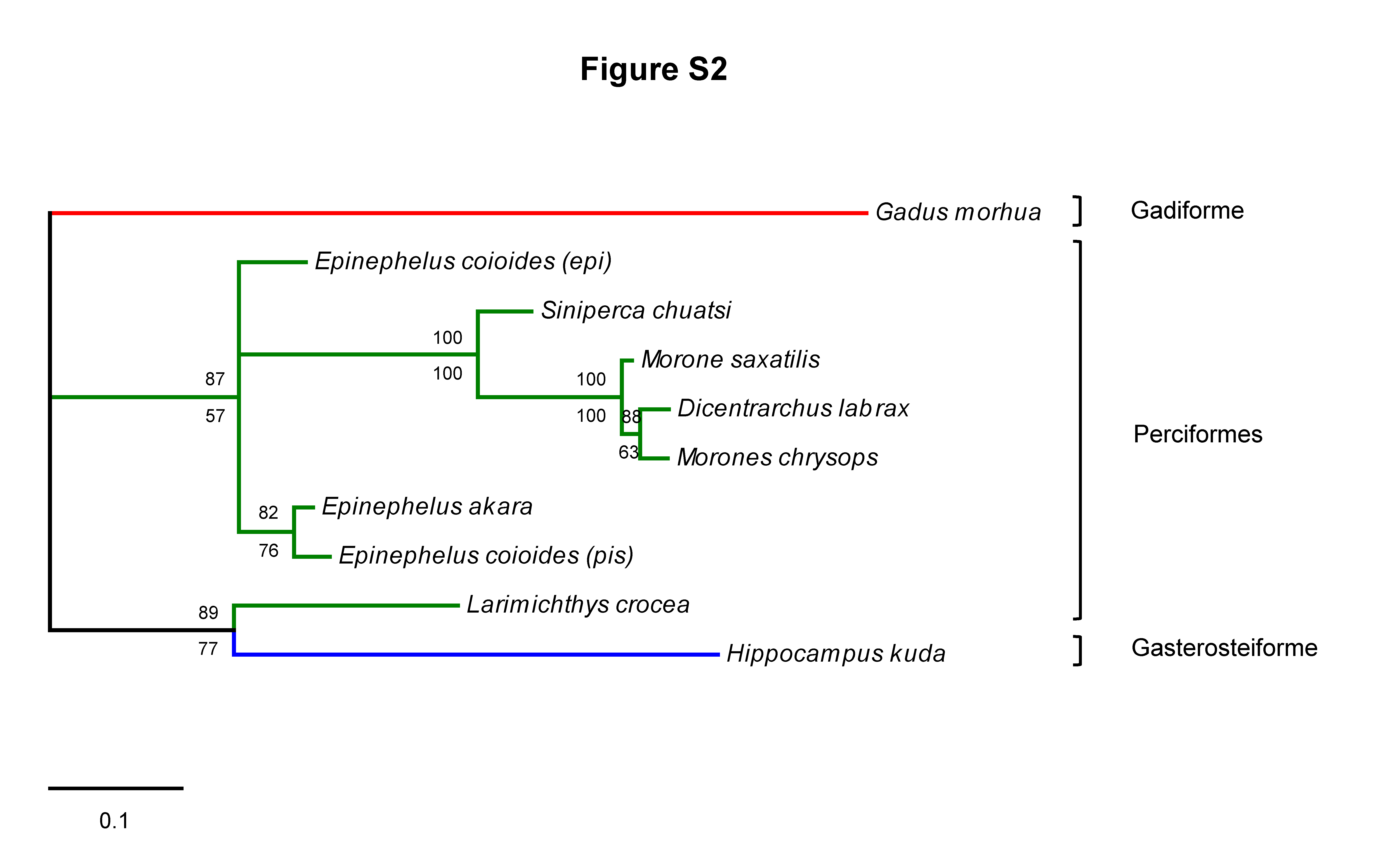

Supplement: Figure S2 — Reconstruction of piscidin phylogeny. This phylogram was constructed using Bayesian and likelihood methods. The SYM+G model was selected for the Bayesian analysis and the consensus tree was built after burning 1,250 trees from the 5•105 generations. The likelihood phylogeny was obtained with a HKY nucleotide substitution model with a discrete γ distribution (4 categories, γ shape parameter 2.0) and 100 bootstrap data sets. Bayesian posterior probabilities and maximum likelihood bootstrap values are indicated as percentages above and below the tree nodes, respectively. The scale bar indicates distance (branch length). (1.04 MB TIF) [file pone.0009501.s004.tif]
